# Comparable Attenuation of Sympathetic Nervous System Activity in Obese Subjects with Normal Glucose Tolerance, Impaired Glucose Tolerance, and Treatment Naïve Type 2 Diabetes following Equivalent Weight Loss

**DOI:** 10.3389/fphys.2016.00516

**Published:** 2016-11-03

**Authors:** Nora E. Straznicky, Mariee T. Grima, Carolina I. Sari, Elisabeth A. Lambert, Sarah E. Phillips, Nina Eikelis, Justin A. Mariani, Daisuke Kobayashi, Dagmara Hering, John B. Dixon, Gavin W. Lambert

**Affiliations:** ^1^Human Neurotransmitters Laboratory, Baker IDI Heart and Diabetes InstituteMelbourne, VIC, Australia; ^2^Department of Physiology, Monash UniversityMelbourne, VIC, Australia; ^3^Department of Physiology, University of MelbourneMelbourne, VIC, Australia; ^4^Heart Failure Research Group, Baker IDI Heart and Diabetes InstituteMelbourne, VIC, Australia; ^5^Faculty of Medicine, Nursing and Health Sciences, Monash UniversityMelbourne, VIC, Australia; ^6^Department of Primary Health Care, Monash UniversityMelbourne, VIC, Australia

**Keywords:** sympathetic nervous system, obesity, weight loss, hypocaloric diet, glucose tolerance, hyperinsulinemia, type 2 diabetes

## Abstract

**Background and Purpose:** Elevated sympathetic nervous system (SNS) activity is a characteristic of obesity and type 2 diabetes (T2D) that contributes to target organ damage and cardiovascular risk. In this study we examined whether baseline metabolic status influences the degree of sympathoinhibition attained following equivalent dietary weight loss.

**Methods:** Un-medicated obese individuals categorized as normal glucose tolerant (NGT, *n* = 15), impaired glucose tolerant (IGT, *n* = 24), and newly-diagnosed T2D (*n* = 15) consumed a hypocaloric diet (29% fat, 23% protein, 45% carbohydrate) for 4-months. The three groups were matched for baseline age (56 ± 1 years), body mass index (BMI, 32.9 ± 0.7 kg/m^2^), and gender. Clinical measurements included whole-body norepinephrine kinetics, muscle sympathetic nerve activity (MSNA, by microneurography), spontaneous cardiac baroreflex sensitivity (BRS), and oral glucose tolerance test.

**Results:** Weight loss averaged −7.5 ± 0.8, −8.1 ± 0.5, and −8.0 ± 0.9% of body weight in NGT, IGT, and T2D groups, respectively. T2D subjects had significantly greater reductions in fasting glucose, 2-h glucose and glucose area under the curve (AUC_0−120_) compared to NGT and IGT (group effect, *P* <0.001). Insulinogenic index decreased in IGT and NGT groups and increased in T2D (group × time, *P* = 0.04). The magnitude of reduction in MSNA (−7 ± 3, −8 ± 4, −15 ± 4 burst/100 hb, respectively) and whole-body norepinephrine spillover rate (−28 ± 8, −18 ± 6, and −25 ± 7%, respectively), time effect both *P* <0.001, did not differ between groups. After adjustment for age and change in body weight, Δ insulin AUC_0−120_ was independently associated with reduction in arterial norepinephrine concentration, whilst Δ LDL-cholesterol and improvement in BRS were independently associated with decrease in MSNA.

**Conclusions:** Equivalent weight loss through hypocaloric diet is accompanied by similar sympathoinhibition in matched obese subjects with different baseline glucose tolerance. Attenuation of hyperinsulinemia and hyperlipidemia, rather than glycemic indices, is associated with reduction in SNS activity following weight loss intervention.

## Introduction

The high prevalence of obesity, estimated to exceed 25% in many developed countries including Australia (Australian Bureau of Statistics, 2015), is a serious public-health concern that confers excess mortality and morbidity due to its associations with chronic conditions such as type 2 diabetes (T2D) and cardiovascular disease (Prospective Studies Collaboration, 2009). It is therefore pertinent to elucidate potential mechanisms that link excess adiposity to cardiometabolic risk and to identify groups most likely to benefit from targeted lifestyle interventions.

The sympathetic nervous system (SNS) plays a pivotal role in both cardiovascular and metabolic regulation and perturbations in SNS activity are widely believed to contribute to the pathophysiology of the obese state. An early viewpoint, based on experimental hypothalamic models of obesity, clinical studies of venous and urinary norepinephrine concentration, and studies in Pima Indians (an ethnic population with low SNS activity) was that SNS underactivity was the basis of weight gain and the development obesity (Bray, 1990; Tataranni et al., 1997; Snitker et al., 2000). This was congruent with the known importance of the SNS in all aspects of daily energy expenditure. However, more recent studies which have applied more specific techniques such as direct microneurographic recordings of efferent sympathetic nerve traffic and isotope dilution, indicate that obesity is a state of chronic sympathetic overactivity and that there is organ specific differentiation in sympathetic outflow favoring skeletal muscle vasculature and the kidneys (Vaz et al., 1997; Grassi et al., 2004, 2005). Elevated SNS activity and impaired baroreflex sensitivity (BRS) is evidenced in obese, metabolic syndrome, and T2D cohorts when compared to lean controls (Lee et al., 2001; Huggett et al., 2003; Grassi et al., 2005) and the greater contribution of visceral, as opposed to subcutaneous fat accumulation, to sympathetic neural drive has been affirmed (Alvarez et al., 2002; Grassi et al., 2004).

Prospective epidemiological and offspring studies provide information on the chronology of pathogenic changes in the general and at risk populations. Various indices of SNS activity (baseline venous norepinephrine concentration, the norepinephrine response to cold pressor test, and resting heart rate) have been positively associated with future weight gain, insulin resistance and hyperglycemia in epidemiological studies with 5–18 years follow-up (Masuo et al., 2003; Flaa et al., 2008; Wulsin et al., 2015). Although seemingly counterintuitive, elevated SNS activity could predispose to weight gain via β-adrenoceptor desensitization and the promotion of central insulin resistance (Vollenweider et al., 1994; Julius et al., 2000). Notably, insulin-sensitive off-spring of T2D patients have elevated single-unit and multi-unit muscle sympathetic nerve activity (MSNA) compared with age- and body mass index (BMI)-matched controls with no family history of T2D (Huggett et al., 2006). This finding highlights that sympathetic overactivity is an early pathogenic phenomenon in subjects genetically predisposed to the development of metabolic disease. In established obesity several factors act in concert to maintain elevated sympathetic drive including hyperinsulinemia, obstructive sleep apnoea, baroreflex impairment, and activation of the renin-angiotensin system (Lambert et al., 2010). Sustained sympathetic activation is mechanistically involved in obesity-related target organ damage such as left ventricular diastolic dysfunction, left ventricular hypertrophy, and arterial remodeling via both blood pressure-mediated and blood pressure-independent effects (Schlaich et al., 2009). Vis-a-vis, plasma norepinephrine is an independent predictor of adverse cerebral and cardiovascular events (Yufu et al., 2014).

Weight loss through energy-restricted diet and/or physical activity represents first-line treatment for obesity and has proven sympathoinhibitory benefits (Straznicky et al., 2010). However, relatively little attention has focused on whether baseline metabolic status modifies the magnitude of change in SNS activity following weight reduction. Our group has previously demonstrated that hyperinsulinemic subjects derive significantly greater sympathoinhibition than normoinsulinemic subjects at matched weight loss (Straznicky et al., 2014). This concurs with the known central sympathoexcitatory effects of insulin (Anderson et al., 1991; Rahmouni et al., 2004). Other studies have shown greater improvement in glycemic and cardiac autonomic indices in T2D subjects compared to normal glucose tolerant (NGT) subjects after lifestyle induced and surgical weight loss (Solomon et al., 2010; Casellini et al., 2016). Hitherto, the relative benefits of weight loss in obese subjects with different stages of glycemic control on robust assessments of SNS activity are unknown. Moreover, there are no data regarding the effects of weight loss on MSNA or norepinephrine kinetics in T2D populations. The present study was conducted to compare the impact of 4-month hypocaloric diet (HCD) on direct recordings of efferent postganglionic MSNA and whole-body norepinephrine kinetics in obese subjects categorized as NGT, impaired glucose tolerant (IGT) and newly diagnosed T2D. We hypothesized that T2D subjects would derive the greatest sympathoinhibition at equivalent weight loss due to greater attenuation of glycemia and concomitant improvement in baroreflex function (Gerritsen et al., 2000).

## Methods

### Subjects

Data were pooled from three weight loss trials conducted in our laboratory (NCT01771042, NCT00163943, and Straznicky et al., 2005). Entry criteria, dietary intervention, and experimental methodologies were identical across the trials. Study participants were recruited from newspaper advertisements on the basis of being a non-smoker, aged 45–65 years, with a BMI > 27 kg/m^2^ and weight stable (±1 kg) in the previous 6 months. Females were post-menopausal. Exclusion criteria comprised history of cardiovascular, cerebrovascular, renal, liver, or thyroid diseases; use of continuous positive airway pressure treatment; and consumption of medications that could affect study parameters (e.g., oral hypoglycemics, cholesterol-lowering, anti-hypertensive, anti-depressant, and hormone replacement therapies). Screening tests were performed over 2 visits 1 week apart. At the first visit written informed consent was obtained and participants' medical and dietary histories were recorded. Clinical testing included an electrocardiogram, fasting biochemistry (urea and electrolytes, liver function tests, and lipids), and supine resting blood pressure, measured as the average of 5 readings after 5 min rest using a Dinamap monitor (Model 1846SX, Critikon Inc., Tampa, FL, USA). Body weight was measured in indoor clothes without shoes, using a digital scale. Waist circumference was measured at its smallest girth and hip circumference at the level of the greater trochanters. Subjects were then given a 4-day prospective diet and exercise diary to be returned the following week. At the second screening visit, diet and exercise records were reviewed, subjects received a physical examination by the study doctor and blood pressure measurements were repeated. Clinical investigations were performed at baseline and after 16-weeks of HCD. The project was approved by the Alfred Hospital Human Research Ethics Committee (ID 1/13).

### Dietary intervention

Habitual dietary intake was assessed by 4-day prospective diet records using Australian food composition tables (Foodworks 7 Professional, Xyris Software, Kenmore Hills Qld 4069). Subjects were prescribed HCD at 25% energy deficit using a modified Dietary Approach to Stop Hypertension (DASH) eating plan (Appel et al., 1997), with higher protein and lower carbohydrate content (25% protein, 30% fat, and 45% carbohydrate) to maximize fat loss (Layman et al., 2003). The DASH diet has an emphasis on fruits, vegetables, nuts, legumes, wholegrain cereals, low-fat dairy foods and lean red meat, fish and poultry. Olive oil and polyunsaturated margarine were the major fat sources. Previous studies have demonstrated that adherence to a DASH dietary pattern during lifestyle intervention is accompanied by beneficial changes in blood pressure, lipid profile and homeostasis model assessment of insulin resistance (HOMA-IR) which are attributed to both weight loss and weight loss-independent effects (Appel et al., 1997; Lien et al., 2007). Participants were provided with written dietary instructions, 14-day menu plans and recipes, and prepared food in their home environment. They attended fortnightly for body weight measurement and dietary counseling with the study dietician. With regards to exercise they were instructed to walk briskly for 30 min, at least 5 days per week, in accordance with the American Diabetes Association Position Statement (Inzucchi et al., 2012). Dietary intake and exercise level was monitored by prospective 4-day records.

### Clinical investigations

Assessments of SNS activity, BRS and oral glucose tolerance were performed together on the same morning. Subjects attended for clinical testing at 8 a.m. after an overnight fast and having abstained from alcohol and heavy exercise for 36 h and caffeine for 18 h. All experiments were carried out in the supine position in a temperature controlled (22°C) research room. Subjects voided prior to commencement.

#### Whole-body norepinephrine kinetics

The dynamic processes of whole-body norepinephrine entry or “spillover” into and removal from the central plasma compartment, were determined under fasting conditions using the isotope dilution method (Esler et al., 1979). This technique involves the intravenous infusion of [^3^H]-norepinephrine and measurement of norepinephrine specific activity and endogenous norepinephrine in arterial blood, sampled from the brachial artery, under steady-state conditions. After a priming bolus of 2.36 μCi of 1-[ring-2,5,6-^3^H]-norepinephrine (Perkin-Elmer, Waltham, MA, US; specific activity, 10–30 μCi/mmol), an infusion was commenced at 0.24 μCi×min^−1^, with arterial blood sampling 30 min after infusion commencement. The following calculations were performed:

$$\begin{array}{l} \text{Norepinephrine clearance}\left(\text{L}/\text{min}\right)=\\ \frac{\left[{\;}^{3}\text{H}\right]-\text{norepinephrine infusion rate}\left(\text{dpm}/\text{min}\right)}{\left[{\;}^{3}\text{H}\right]-\text{norepinephrine plasma concentration}\left(\text{dpm}/\text{ml}\right)\;\times \text{ 1}000}\\ \text{Norepinephrine spillover}\left(\text{ng}/\text{min}\right)=\\ \frac{\text{plasma norepinephrine}\left(\text{pg}/\text{ml}\right)\;\times \;\text{clearance}\left(\text{ml}/\text{min}\right)}{1000} \end{array}$$

#### Muscle sympathetic nerve activity

Recordings of fasting multi-unit postganglionic MSNA were made from a tungsten microelectrode (FHC, Bowdoinham, ME, USA) inserted into the right peroneal nerve at the fibular head (Vallbo et al., 2004). A subcutaneous reference electrode was positioned 2–3 cm away from the recording site. Standard criteria were used to ascertain a MSNA site. The nerve signal was amplified (X50,000), filtered (bandpass, 700–2000 Hz), and integrated. Intra-arterial blood pressure, ECG, respiration, and MSNA were digitized with a sampling frequency of 1000 Hz (PowerLab recording system, model ML 785/8SP, ADI Instruments). Resting measurements were recorded over a 15-min period and averaged. Sympathetic bursts were counted manually and expressed as burst frequency (bursts/min) and burst incidence (bursts/100 heart beats).

#### Cardiac baroreflex sensitivity

Spontaneous cardiac BRS was assessed by the sequence method (Parati et al., 2004). The slope of the regression line between cardiac interval and systolic blood pressure was calculated for validated sequences of three or more consecutive heartbeats in which systolic blood pressure progressively increased and cardiac interval lengthened (type 1 sequence) or systolic blood pressure progressively decreased and cardiac interval shortened (type 2 sequence), with a lag of 1 beat. Recordings were averaged over a 15-min period of supine rest.

#### Metabolic and biochemical measurements

After completion of norepinephrine kinetic and microneurographic measurements subjects underwent a standard OGTT. It is of note that high specific activity [^3^H]-norepinephrine has no physiological effects at the doses used (Esler et al., 1979). A 2-h 75-g oral glucose tolerance test (OGTT, Glucaid, Fronine PTY, LTD, Taren Point, NSW 2229, Australia) was administered with 30-min intravenous blood sampling for determination of glucose and insulin responses (area under the curve, AUC_0−120_) using the trapezoidal rule. Homeostasis model assessment-insulin resistance (HOMA-IR) and the Matsuda insulin sensitivity index (ISI) were calculated as indices of insulin resistance and sensitivity, respectively (Matthews et al., 1985; Matsuda and DeFronzo, 1999). The insulinogenic index (Δ insulin 0–30 min divided by Δ glucose 0–30 min) was used to assess pancreatic β-cell function. Categorization was based on WHO diagnostic criteria (World Health Organization, 2006): T2D, fasting plasma glucose ≥7.0 mmol/L or 2-h plasma glucose ≥11.1 mmol/L; IGT, fasting plasma glucose <7.0 mmol/L and 2-h plasma glucose ≥7.8 and <11.1 mmol/L; NGT, 2-h plasma glucose <7.8 mmol/L. Supine fasting blood samples were obtained for measurement of lipid profile, non-esterified fatty acids (NEFAs), high sensitivity C-reactive protein (hs-CRP), leptin, and plasma renin activity (PRA).

### Laboratory assays

Plasma glucose and lipid profile were measured on a commercial analytical system (Architect C18000 analyzer, Abbott Laboratories, Illinois, USA). High sensitivity C-reactive protein (*hs*-CRP) was quantified by immunoturbidimetric assay, insulin, leptin, and PRA by radio-immuno assay (Linco Research, Inc., Missouri, USA; REN-CT2, CIS bio international, France) and NEFA by enzymatic colorimetry (Wako Pure Chemical Industries, Ltd, Osaka, Japan). These assays were performed in duplicate. Plasma norepinephrine was assayed by high performance liquid chromatography with electrochemical detection, following extraction by alumina adsorption. [^3^H]-norepinephrine was assayed by liquid scintillation chromatography and corrected for loss during extraction using recoveries of internal standard. Intra-assay CVs in our laboratory are 1.3% for norepinephrine and 2.3% for [^3^H]-norepinephrine; inter-assay CVs are 3.8 and 4.5%, respectively.

### Statistical analyses

Statistical analysis was performed using SigmaStat Version 3.5 (Systat Software Inc., Point Richmond, CA, USA). Data are presented as the mean ± SEM. Baseline parameters in the three study groups were compared by one-way ANOVA and Kruskal-Wallis one-way ANOVA on ranks. Two-way repeat measures ANOVA was used to test for group effects, time effects and group × time interactions, with the Holm-Sidak test for multiple *post-hoc* comparisons. Non-parametric data were log transformed. Post-intervention changes in study parameters (Δ) were compared by one-way ANOVA and one-way ANOVA on ranks as appropriate. Sub-group analysis according to baseline hyperinsulinemia (defined as insulin AUC_0−120_ > 9500 mU · min · L^−1^, the median value in the cohort) was performed by 2-way ANOVA. Univariate associations between change in SNS activity and other variables were assessed using Pearson's correlations in the pooled data set. Forward stepwise linear regression analysis, adjusted for age and change in body weight, was performed to identify the independent predictors of change SNS parameters. Variables with *P* ≤ 0.05 in univariate analyses were entered into the regression model. Statistical significance was accepted at the *P* <0.05 (two-tailed) level.

## Results

### Subjects

Table 1 presents baseline demographic, anthropometric, and clinical data of the study participants. The groups were matched for age, BMI, lipid profile, and clinic blood pressure. Each group exercised for ~30 min per day, primarily in the form of walking. In accordance with the categorization, T2D subjects had higher fasting glucose, 2-h glucose, glucose AUC_0−120_ and HOMA-IR and lower insulinogenic index compared to NGT and IGT groups. The insulin response to OGTT was greatest in the NGT, albeit differences between groups did not reach statistical significance (*P* = 0.12). Baseline measures of SNS activity did not differ between study groups. Arterial norepinephrine concentration averaged 333 ± 49, 274 ± 24, and 258 ± 25 pg/ml in NGT, IGT, and T2D groups, respectively (*P* = 0.35). Corresponding values for whole-body norepinephrine spillover rate were 701 ± 95, 625 ± 68, and 563 ± 76 ng/min (*P* = 0.42) and MSNA burst incidence were 63 ± 5, 65 ± 3 and 64 ± 4 bursts/100 hb (*P* = 0.93).

**Table 1:** **Demographic and clinical data at baseline**.

	**NGT (*n* = 15)**	**IGT (*n* = 24)**	**T2D (*n* = 15)**	***P***
Age (yrs)	56 ± 2	57 ± 1	56 ± 2	0.88
Gender (M/F)	9/6	13/11	6/9	0.52
Body weight (kg)	96.4 ± 3.4	96.8 ± 2.6	97.8 ± 4.0	0.96
Body mass index (kg/m^2^)	32.0 ± 0.8	33.1 ± 0.8	33.6 ± 1.9	0.64
Waist circumference (cm)	110.3 ± 2.6	107.4 ± 2.2	106.7 ± 2.8	0.61
Waist: Hip ratio	0.96 ± 0.02	0.92 ± 0.02	0.91 ± 0.02	0.22
Exercise (min/day)	36 ± 8	31 ± 6	35 ± 9	0.86
Fasting glucose (mmol/L)	5.5 ± 0.1^***^	5.6 ± 0.1^***^	6.7 ± 0.2	<0.001
2-hour glucose (mmol/L)	6.5 ± 0.3^***^^‡^	9.4 ± 0.2^***^	13.2 ± 0.7	<0.001
Glucose AUC_0−120_(mmol · min · L^−1^)	1035 ± 45^***^^†^	1167 ± 33^***^	1489 ± 44	<0.001
Fasting insulin (mU/L)	13.8 ± 1.7	16.3 ± 1.2	18.5 ± 1.5	0.12
Insulin AUC_0−120_(mU · min · L^−1^)	11761 ± 981	9606 ± 798	8819 ± 1104	0.12
HOMA-IR	3.56 ± 0.49^**^	4.14 ± 0.30^*^	5.47 ± 0.50	0.01
Matsuda ISI	2.68 [1.88, 3.49]	2.29 [1.88, 2.87]	1.93 [1.35, 2.48]	0.08
Insulinogenic index (mU insulin · mmol^−1^ glucose)	22.5 ± 3.1^***^^†^	15.9 ± 1.7^*^	9.3 ± 1.5	0.001
LDL-cholesterol (mmol/L)	3.5 ± 0.3	3.5 ± 0.2	3.4 ± 0.2	0.81
HDL-cholesterol (mmol/L)	1.23 ± 0.07	1.21 ± 0.04	1.20 ± 0.07	0.92
Triglycerides (mmol/L)	1.4 [1.2, 2.1]	1.3 [1.1, 2.0]	1.3 [1.2, 1.7]	0.71
Hs-CRP (mg/L)	2.5 [1.5, 2.9]	2.3 [1.2, 3.6]	1.7 [1.0, 4.6]	0.95
NEFA (mEq/L)	0.51 ± 0.05	0.54 ± 0.04	0.59 ± 0.05	0.52
Leptin (ng/ml)	10 [7, 23]	15 [9, 26]	17 [7, 37]	0.69
Systolic BP (mmHg)	129 ± 4	126 ± 3	134 ± 5	0.29
Diastolic BP (mmHg)	75 ± 2	71 ± 2	75 ± 3	0.35
Heart rate (bpm)	65 ± 2	62 ± 2	63 ± 2	0.72
Cardiac BRS (msec/mmHg)	10.2 [7.9, 16.1]	11.5 [7.3, 14.9]	11.9 [8.3, 13.7]	0.99
PRA (ng/ml/h)	0.88 [0.55, 1.32]	0.54 [0.32, 0.89]	0.61 [0.26, 1.17]	0.20

*Values are mean ± SEM or median [interquartile range]. AUC, area under the curve; BP, blood pressure; BRS, baroreflex sensitivity; HDL, high-density lipoprotein; HOMA-IR, homeostasis model assessment-insulin resistance; hs-CRP, high-sensitivity C-reactive protein; ISI, insulin sensitivity index; LDL, low-density lipoprotein; NEFA, non-esterified fatty acids; PRA, plasma renin activity*.

*** *P <0.001*,

** P <0.01 and

* P <0.05 vs. T2D group;

† P <0.05 and

‡ *P <0.01 vs. IGT group*.

### Anthropometrics and dietary intake

Weight loss averaged −7.9 ± 0.4% of body weight overall (range −2.1 to −14.0%) and the magnitude of change did not differ between groups (Table 2). Concomitantly, waist circumference decreased by −7.3 ± 0.5 cm. Compared with subjects' baseline diet records, during the intervention total caloric intake decreased by −559 ± 54 kcal/day, total fat by −2.6 ± 0.7% of energy, saturated fat by −2.0 ± 0.5% of energy, sodium by −578 ± 146 mg/day, and protein intake increased by 3.9 ± 0.6% of energy (P all <0.001). There were no significant between group differences in dietary parameters. Time spent exercising increased by 14 ± 4 min/day in the NGT (*P* = 0.04), 12 ± 5 min/day in the IGT (*p* = 0.02), and by 0 ± 7 min/day in the T2D groups (*P* = 0.94).

**Table 2:** **Changes in selected anthropometric and clinical data after hypocaloric diet**.

**Parameter**	**NGT (*n* = 15)**	**IGT (*n* = 24)**	**T2D (*n* = 15)**	**Time effect (P)**	**Time × Group (P)**
**ANTHROPOMETRICS**					
Δ Body weight (kg)	−7.2 ± 0.7^***^	−7.8 ± 0.5^***^	−7.9 ± 1.0^***^	<0.001	0.75
Δ Body weight (%)	−7.5 ± 0.8^***^	−8.1 ± 0.5^***^	−8.0 ± 0.9^***^	<0.001	0.79
Δ Body mass index (kg/m^2^)	−2.4 ± 0.2^***^	−2.7 ± 0.2^***^	−2.6 ± 0.3^***^	<0.001	0.62
Δ Waist circumference (cm)	−7.6 ± 1.2^***^	−7.3 ± 0.6^***^	−7.0 ± 1.1^***^	<0.001	0.92
Δ Waist: hip ratio	−0.03 ± 0.01^**^	−0.03 ± 0.01^***^	−0.02 ± 0.01	<0.001	0.62
**METABOLIC AND BIOCHEMICAL**					
Δ Glucose AUC_0−120_ (mmol · min · L^−1^)	−94 ± 32^*^^†^	−15 ± 22^‡^	−225 ± 49^***^	<0.001	<0.001
Δ Fasting insulin (mU/L)	−3.3 ± 0.7^***^	−3.6 ± 0.7^***^	−4.4 ± 1.2^***^	<0.001	0.72
Δ HOMA-IR	−0.99 ± 0.21^**^	−1.01 ± 0.19^***^	−1.70 ± 0.47^***^	<0.001	0.17
Δ Matsuda ISI	1.81 ± 0.63^***^	0.65 ± 0.13^***^	1.30 ± 0.33^***^	<0.001	0.053
Δ Matsuda ISI (%)	60 ± 16^#^	30 ± 6	61 ± 13^#^	<0.001	0.06
Δ LDL-cholesterol (mmol/L)	−0.3 ± 0.1^*^	−0.2 ± 0.1^*^	−0.3 ± 0.1^*^	<0.001	0.91
Δ HDL-cholesterol (mmol/L)	−0.02 ± 0.05	0.05 ± 0.03	−0.03 ± 0.05	0.99	0.33
Δ Triglycerides (mmol/L)	−0.4 ± 0.2	−0.5 ± 0.1^***^	−0.4 ± 0.1^***^	<0.001	0.40
Δ NEFA (mEq/L)	−0.04 ± 0.04	−0.02 ± 0.03	−0.05 ± 0.05	0.11	0.81
Δ Leptin (ng/ml)	−5.1 ± 0.9^***^	−6.7 ± 1.1^***^	−9.2 ± 2.8^***^	<0.001	0.69
Δ hs-CRP (mg/L)	−0.5 ± 0.2^**^	−0.9 ± 0.3^***^	−0.3 ± 0.3	<0.001	0.24
Δ PRA (ng/ml/h)	−0.36 ± 0.18	−0.01 ± 0.10	−0.17 ± 0.30	0.59	0.36

*Values are mean ± SEM*.

*** *P ≤ 0.001*,

** P ≤ 0.01 and

* P < 0.05 vs. baseline;

† P < 0.05 and

‡ P < 0.01 vs. T2D;

# *P < 0.05 vs. IGT*.

### Glucose metabolism and insulin action

**Figure 1:**
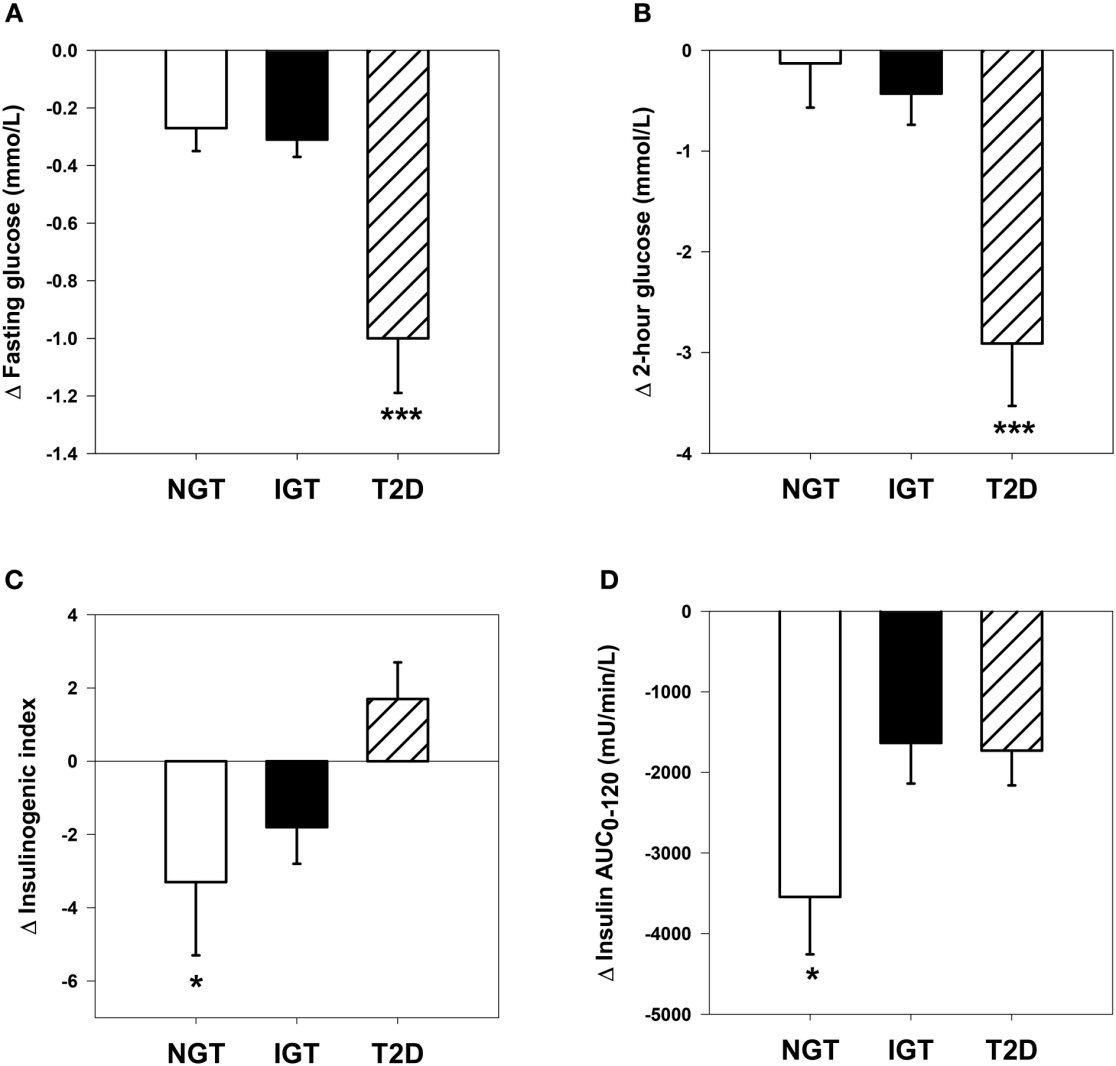
**Comparative changes in glucose and insulin parameters. (A)** Fasting plasma glucose: time effect, *P* <0.001; time × group interaction, *P* <0.001. ^***^P <0.001 vs. NGT and IGT groups. **(B)** Two-hour plasma glucose following oral glucose tolerance test: time effect, *P* <0.001; time × group interaction, *P* <0.001. ^***^P <0.001 vs. NGT and IGT groups. **(C)** Insulinogenic index: time × group interaction, *P* = 0.04. ^*^P <0.05 vs. T2D group. **(D)** Plasma insulin area under the curve during oral glucose tolerance test: time effect, *P* <0.001; time × group interaction, *P* = 0.04. ^*^P <0.05 vs. IGT and T2D groups.

Figure 1 shows comparative changes in glucose and insulin parameters following HCD. The obese T2D group experienced significantly greater reductions in fasting glucose, 2-h glucose and glucose AUC_0−120_, compared with the NGT and IGT groups (group effect, P all <0.001). Fasting insulin and the insulin AUC_0−120_ were significantly reduced after HCD in all three groups (P all <0.01), however the magnitude of change in the latter was greater in the NGT group vs. IGT and T2D groups (P both <0.05). The insulinogenic index decreased in NGT and IGT groups, but increased in the T2D group (group × time, *P* = 0.04). Matsuda ISI increased significantly in all groups (P all <0.001) with the magnitude of change tending to be greater in NGT and T2D group vs. IGT (*P* = 0.053, Table 2).

### Blood chemistry

There were significant reductions in plasma leptin, LDL-cholesterol, triglycerides, and hs-CRP after the HCD intervention, with no significant group by time interactions (Table 2). Plasma NEFA, HDL-cholesterol, and PRA were unchanged in all three groups.

### Sympathetic neural activity

**Figure 2:**
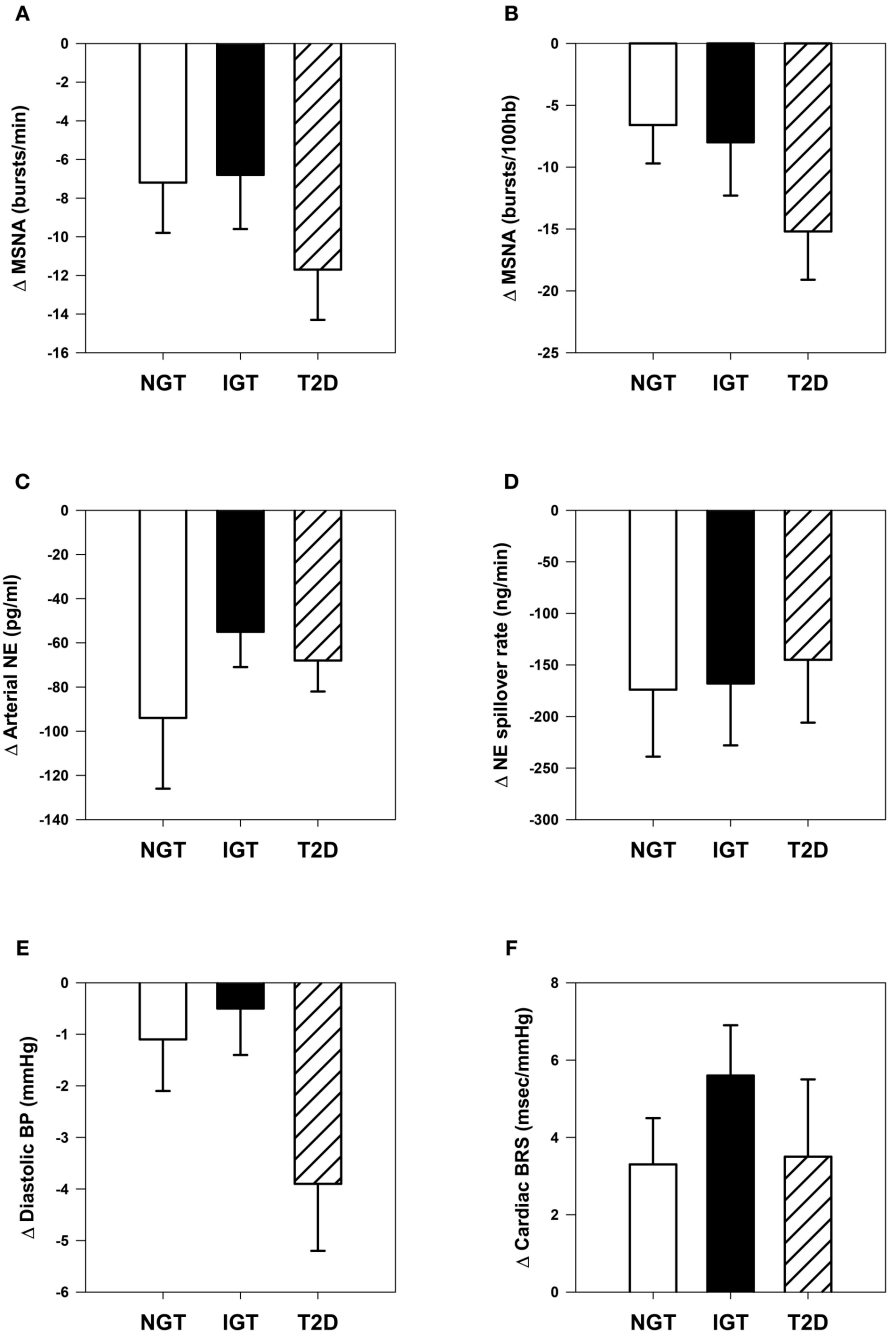
**Comparative changes in sympathetic neural and cardiovascular parameters. (A)** Muscle sympathetic nerve activity (MSNA) expressed as burst frequency: time effect, *P* <0.001. **(B)** MSNA expressed as burst incidence: time effect, *P* <0.001. **(C)** Arterial norepinephrine (NE) concentration: time effect, *P* <0.001. **(D)** Whole-body NE spillover rate: time effect, *P* <0.001. **(E)** Clinic diastolic blood pressure (BP): time effect, *P* = 0.005; time × group interaction, *P* = 0.09. **(F)** Spontaneous cardiac baroreflex sensitivity (BRS): time effect, *P* <0.001.

Acceptable paired microneurographic recordings were obtained in 43 subjects (12 NGT, 19 IGT, and 12 T2D). MSNA was significantly attenuated when expressed as burst frequency or burst incidence with no significant between group differences (Figures 2A,B).

Arterial norepinephrine concentration decreased significantly in each group (Figure 2C, P all ≤ 0.001) by −23 ± 6, −16 ± 4, and −25 ± 4% in NGT, IGT, and T2D groups respectively (between group *P* = 0.39). Norepinephrine plasma clearance was unchanged in all three groups. Calculated whole-body norepinephrine spillover rate decreased in all groups, but the magnitude of change did not differ (Figure 2D).

**Figure 3:**
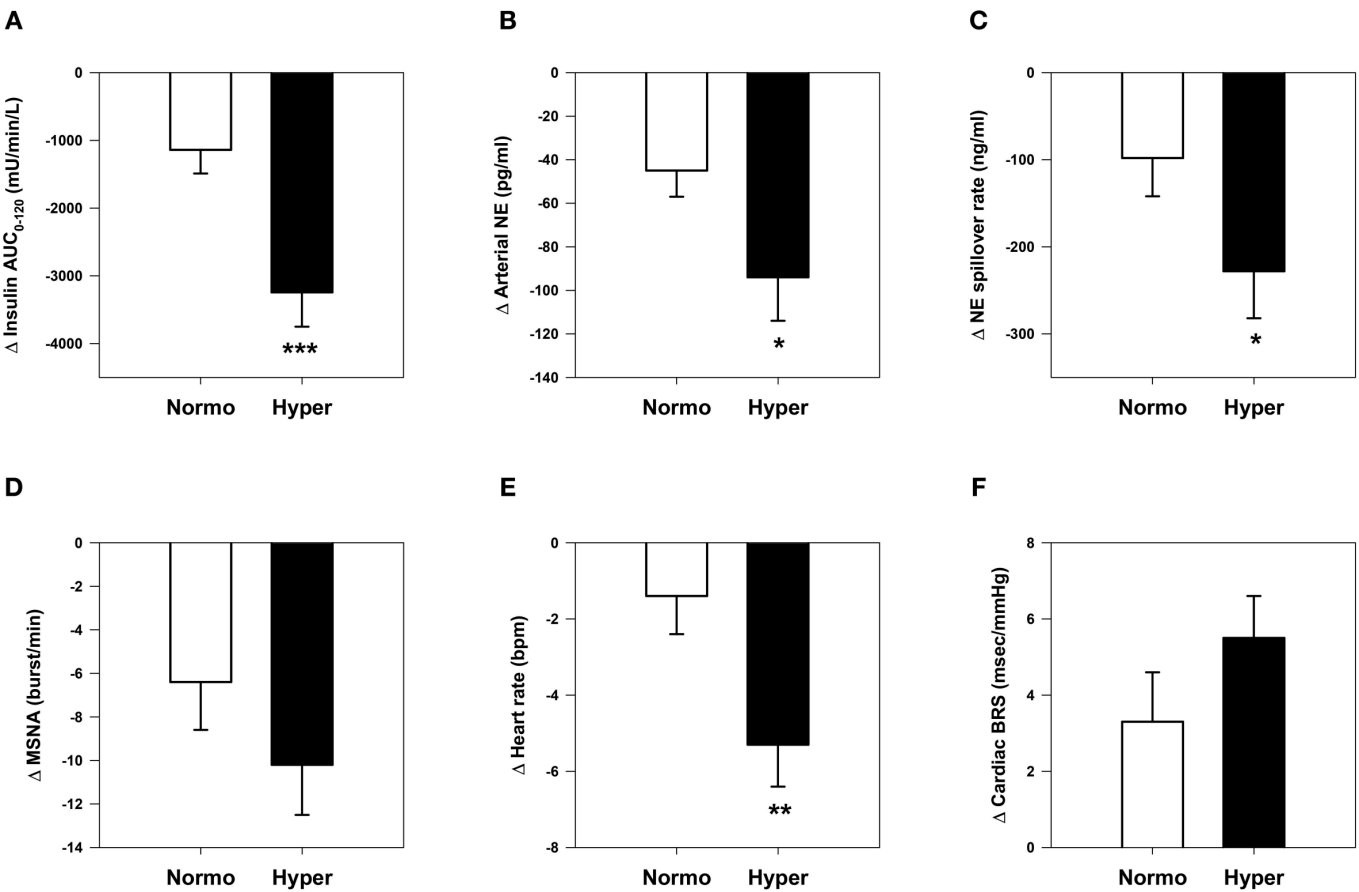
**Comparative changes in sympathetic neural and cardiovascular parameters in normoinsulinemic and hyperinsulinemic subjects. (A)** Insulin area under the curve (AUC) during oral glucose tolerance test. **(B)** Arterial plasma norepinephrine (NE) concentration. **(C)** Whole-body NE spillover rate. **(D)** Muscle sympathetic nerve activity (MSNA) burst frequency. **(E)** Heart rate. **(F)** Spontaneous cardiac baroreflex sensitivity (BRS). ^*^P <0.05, ^**^P <0.01, and ^***^P <0.001 vs. normoinsulinemic group. Normoinsulinemic: insulin AUC_0−120_ ≤ 9500 mU/min/L (*n* = 27); hyperinsulinemic: insulin AUC_0−120_ > 9500 mU/min/L (*n* = 27).

Subgroup analysis by baseline hyperinsulinemia (Figure 3) showed that hyperinsulinemic subjects (baseline insulin AUC_0−120_, 13,374 ± 498 mU · min · L^−1^) experienced significantly greater reductions in insulin AUC_0−120_, arterial norepinephrine concentration, whole-body norepinephrine spillover rate, and heart rate than normoinsulinemic counterparts (baseline insulin AUC_0−120_, 6598 ± 352 mU · min · L^−1^), despite similar weight loss (−7.7 ± 0.6 and −7.6 ± 0.6 kg, respectively). Hyperinsulinemic subjects had significantly lower BRS at baseline compared with normoinsulinemic subjects (11.1 ± 1.3 vs. 15.0 ± 1.7 ms/mmHg, *P* = 0.03).

### Cardiovascular parameters

Clinic systolic blood pressure decreased by −7 ± 2, −5 ± 2, and −9 ± 3 mmHg in NGT, IGT, and T2D groups respectively (time effect, *P* <0.001; group effect, *P* = 0.46). Clinic diastolic blood pressure decreased significantly only in the T2D group (time effect, *P* <0.01; group effect *P* = 0.09, Figure 2E). Spontaneous BRS was enhanced in all three groups (time effect *P* <0.001), with no significant differences between groups (Figure 2F).

### Regression analyses

Reduction in sympathetic neural parameters was not associated with changes in body weight, body mass index, waist circumference, glycemic indices, or dietary parameters. Stepwise regression analyses of the pooled data set showed that reduction in insulin AUC_0−120_ was independently associated with change in arterial norepinephrine concentration, explaining 9% of the variance (*P* = 0.03, Table 3). This relationship was stronger in males, where Δ insulin AUC_0−120_ explained 49% of the variance (*P* <0.001) in norepinephrine levels following HCD. Change in whole-body norepinephrine spillover rate was significantly associated with changes in plasma leptin level and PRA, whilst attenuation in MSNA related to improvement in BRS and reduction in LDL-cholesterol (Table 3). Improvement in BRS was most strongly correlated with reduction in triglyceride levels (*r* = −0.36, *P* = 0.01).

**Table 3:** **Stepwise regression analyses of changes in sympathetic neural parameters following hypocaloric diet**.

**Dependent variables**	**Step**	**Independent variables**	**Std. coeff**	***r*^2^**	***P***
Δ Log NE (pg/ml)	1	Δ Insulin AUC_0−120_ (mU/min/L)	0.30	0.09	0.03
Δ Log NE spillover rate (ng/min)	1	Δ Leptin (ng/ml)	0.33	0.13	0.01
	2	Δ PRA (ng/ml/h)	0.29	0.21	0.03
Δ MSNA (bursts/min)	1	Δ BRS (msec/mmHg)	−0.31	0.15	0.046
	2	Δ LDL-C (mmol/L)	0.30	0.23	0.049
Δ MSNA (bursts/100hb)	1	Δ LDL-C (mmol/L)	0.33	0.11	0.04

*Regression analyses were adjusted for age (years), change in body weight (kg) and change in exercise (min/day). MSNA, muscle sympathetic nerve activity; NE, norepinephrine*.

## Discussion

In this study we sought to compare the benefits of equivalent weight loss, achieved through HCD, on SNS activity in un-medicated, matched obese subjects who were stratified according to their glucose tolerance, and who are representative of distinct metabolic sub-groups along the diabetes continuum. In accordance with previously published data, we found that T2D subjects experienced greater reductions in fasting and post-OGTT glucose levels and an increase in insulinogenic index compared with their non-diabetic counterparts (Solomon et al., 2010). However, the greater improvement in glycemic indices did not translate to any additive effect on spontaneous cardiac BRS nor on sympathoinhibition. Indeed, weight loss was associated with significant and similar attenuation in both neurophysiological and biochemical measures of sympathetic activity across the groups. To our knowledge, ours is the firsts study to demonstrate that moderate weight loss attenuates MSNA and whole-body norepinephrine spillover rate in treatment naïve T2D subjects. Pooled regression analysis indicated that reductions in hyperinsulinemia, gauged as the insulin response during OGTT, leptin and PRA, and enhancement of BRS were independently associated with sympathoinhibition within the cohort. These findings are elaborated below.

Only one other study has compared weight loss associated improvements in autonomic function in different obese subgroups. Casellini et al. (2016) measured time and frequency domain analysis of heart rate variability before and after surgical weight loss in non-diabetic, pre-diabetic, and T2D subjects. They reported that changes in cardiac autonomic function, representing both sympathetic and parasympathetic components, were significantly greater in T2D subjects. However, this study is not comparable to ours because of differences in participants' clinical status (their T2D subjects had long-standing drug-treated diabetes and non-diabetic subjects were normoinsulinemic); the mode and magnitude of weight loss (four times greater than that attained in our study); and the indirect quantification of cardiac autonomic tone. In contrast, our NGT group was hyperinsulinemic and we found no differentiation in sympathetic neural activity or spontaneous cardiac BRS at baseline, nor in the response to HCD, between our three groups. Previous cross-sectional studies have shown higher MSNA and lower BRS in T2D subjects vs. matched normoinsulinemic NGT subjects (Gerritsen et al., 2000; Huggett et al., 2003). This discrepancy is likely explained by the hyperinsulinemic status of our NGT group and our findings need to be interpreted in this context. Furthermore, categorization as NGT in our study was based only on 2-h glucose concentration, thus some NGT subjects may have had impaired fasting glucose, which could have influenced the results by minimizing between-group differences. Albeit, a recent study reported no differences in BRS between subjects categorized as having isolated impaired fasting glucose vs. those with NGT (Wu et al., 2014).

There is now ample clinical and experimental evidence that links hyperinsulinemia to augmented central sympathetic outflow (Anderson et al., 1991; Rahmouni et al., 2004). Distinct central signaling pathways have been identified that mediate insulin-induced regional sympathoactivation to skeletal muscle, adipose tissue and the kidneys (Rahmouni et al., 2003, 2004). In the present study, reduction in hyperinsulinemia was independently associated with attenuation in arterial norepinephrine concentration, which concurs with our previous findings in other cohorts (Straznicky et al., 2014). This observation also concurs with the demonstration that individuals with higher baseline plasma insulin derive the greatest improvement in macrovascular and microvascular endothelial function following weight loss (Bigornia et al., 2013). Furthermore, *post-hoc* analyses in the Swedish Obese Study indicated that higher baseline insulin concentration was associated with a more favorable outcome of bariatric surgery on cardiovascular events after a median follow-up of 15 years (Sjöström et al., 2012). Aside from its direct effects on central sympathetic outflow, hyperinsulinemia secondary to insulin resistance may also modulate BRS by both peripheral and central mechanisms. Peripherally, insulin resistance and attendant pathophysiologies (glucotoxicity, lipotoxicity, and inflammation) promote endothelial dysfunction, arterial stiffening, and impaired baroreceptor afferent responses to arterial wall stretch (Watkins et al., 2000; Grassi et al., 2005; Kim et al., 2006). In the brain, insulin increases the gain of baroreflex control of heart rate and MSNA (Pricher et al., 2008; Young et al., 2010). Therefore, central insulin resistance which is a characteristic of the metabolic syndrome, may reverse the normal effects of brain insulin to support optimal baroreflex control (Kaiyala et al., 2000). Consistent with these mechanisms, hyperinsulinemic subjects had lower baseline BRS compared with normoinsulinemic subjects in our study. It is of note that the observed dichotomy of changes in insulinogenic index between T2D and NGT subjects after HCD has been reported before. Solomon et al. (2010) demonstrated improved insulin secretion in T2D subjects following moderate dietary weight loss vs. reversal of compensatory hyperinsulinemia in nondiabetic individuals. Relief of extreme hyperglycemia (glucotoxicity) and enhanced secretion of the incretin hormone, glucose-dependent insulinotropic polypeptide, explained the improvement in pancreatic β-cell function in T2D subjects in their study.

Other factors that predicted sympathoinhibition in our study included reductions in plasma leptin, LDL-cholesterol, and activity of the renin-angiotensin system. In addition to its classic effects on appetite, the adipokine leptin is known to increase regional sympathetic nerve activity to the kidneys, adrenal gland, hindlimb, and adipose tissue in laboratory animals (Mark, 2013). The evidence for leptin-induced sympathoexcitation in humans is less strong, and is limited to correlative data, including our own showing an association with renal norepinephrine spillover rate in healthy men of differing adiposity (Eikelis et al., 2003) and the fact that leptin deficiency states are associated with sympathetic hypofunction (Ozata et al., 1999). Clinical leptin administration studies have yielded equivocal results (Mackintosh and Hirsch, 2001; Machleidt et al., 2013). A bidirectional interaction between the SNS and the renin-angiotensin-aldosterone system (RAAS) has been long appreciated (Grassi, 2001). RAAS activation in obesity has been attributed to increased synthesis of angiotensinogen by visceral fat, renal compression, and increased sympathetic outflows to the kidneys stimulating renin release. Elevated plasma LDL-cholesterol concentration is an established trigger for impairment of endothelial function and we have previously demonstrated both elevated MSNA and diminished endothelial function in young adults with hypercholesterolaemia compared to normal controls (Lambert et al., 2013). In the present study LDL-cholesterol decreased significantly in all three groups, likely in response to the reduced saturated fat content of the background diet which averaged −10 g per day, and changes in LDL independently related to reduction in MSNA.

Our study has a number of limitations which need to be noted. Firstly, it did not include a control arm, however we have previously demonstrated the reproducibility of MSNA and norepinephrine kinetics measurements in weight stable subjects over a 3-month period (Straznicky et al., 2010). Moreover, any effects of familiarization on sympathetic neural measurements would have been similar across the groups. Secondly, we did not assess body composition using a robust technique such as dual energy x-ray absorptiometry, so the impact of changes in fat depots on SNS parameters could not be determined. Thirdly, the study does not distinguish the effects of weight loss vs. negative energy balance from dietary restriction. Fourthly, subject numbers were relatively small and we may not have had adequate power to detect small differences between groups. Finally, we did not have any robust measures of fitness in order to ascertain whether changes in exercise habit contributed to sympathoinhibition. Exercise diaries indicated that on average our cohort walked 30 min per day at baseline, and that the NGT and IGT groups increased their average walking times by 12–14 min per day, whereas there were no increments in the T2D group during the intervention. Aerobic exercise has independent beneficial effects on SNS activity, endothelial function, baroreflex modulation, and insulin sensitivity, which may have contributed to our findings (Jennings et al., 1986). Results of the CALERIE study indicate that at identical energy deficit incorporation of exercise training with caloric restriction augmented improvements in cardiac sympathovagal balance compared to calorie restriction alone (deJonge et al., 2010).

In summary, our study demonstrates that moderate weight loss following HCD significantly attenuates SNS activity in obese subjects with diverse glycemic status. Concurrent reductions in hyperinsulinemia, leptin, LDL-cholesterol, and RAS were associated with the sympathoinhibition attained. These findings are of clinical importance in the context that elevated SNS activity contributes to the pathogenesis of hypertension, target organ damage and adverse cardiovascular and metabolic outcomes in both non-obese and obese populations (Masuo et al., 2003; Flaa et al., 2008; Schlaich et al., 2009). Additional studies are needed to address (1) the benefits of weight loss on autonomic function in hypoinsulinemic individuals with T2D; (2) the length of time that reduced sympathetic neural function is maintained after weight loss; and (3) the relationship between glycemic variability assessed by 24-h continuous monitoring and SNS activity.

## Author contributions

NS, EL, JD, and GL conceived the study and contributed to the interpretation of the data. SP and NE performed laboratory analyses. NS, MG, CS, EL, DK, DH, and JM collected clinical data. NS performed statistical analysis and wrote the manuscript. All authors read and had final approval of the manuscript.

## Funding

This study was funded by a Diabetes Australia Millennium Grant in type 2 diabetes to NS. JD and GL are supported by NHMRC Fellowships. We also wish to acknowledge the Victorian Government's Operational Infrastructure Support Program.

### Conflict of interest statement

NS, MG, CS, EL, SP, NE, DK, DH declare that the research was conducted in the absence of any commercial or financial relationships that could be construed as a potential conflict of interest. JD is a consultant for Apollo Endosurgical, Bariatric Advantage, and is a member of the Optifast® Medical Advisory Board for Nestle Health, Australia and the Saxenda Advisory Board for Novo Nordisk. JM has received research grants and teaching honoraria from Medtronic. GL has acted as a consultant for Medtronic and has received honoraria from Medtronic, Pfizer, and Wyeth Pharmaceuticals for presentations. These organizations played no role in the design, analysis or interpretation of data described here, nor in the preparation, review, or approval of the manuscript.

## References

[B1] Alvarez G. E. Beske S. D. Ballard T. P. Davy K. P. (2002). Sympathetic neural activation in visceral obesity. Circulation 106, 2533–2536. 10.1161/01.CIR.0000041244.79165.25 12427647

[B2] Anderson E. A. Hoffman R. P. Balon T. W. Sinkey C. A. Mark A. L. (1991). Hyperinsulinemia produces both sympathetic neural activation and vasodilation in normal humans. J. Clin. Invest. 87, 2246–2252. 10.1172/JCI115260 2040704 PMC296986

[B3] Appel L. J. Moore T. J. Obarzanek E. Vollmer W. M. Svetkey L. P. Sacks F. M.. (1997). A clinical trial of the effects of dietary patterns on blood pressure. N. Engl. J. Med. 336, 1117–1124. 10.1056/NEJM199704173361601 9099655

[B4] Bigornia S. J. Farb M. G. Tiwari S. Karki S. Hamburg N. M. Vita J. A.. (2013). Insulin status and vascular responses to weight loss in obesity. J. Am. Coll. Cardiol. 62, 2297–2305. 10.1016/j.jacc.2013.07.078 23978693 PMC3873767

[B5] Bray G. A. (1990). Obesity- A state of reduced sympathetic activity and normal or high adrenal activity (The autonomic and adrenal hypothesis revisited). Int. J. Obesity 14(Suppl. 3), 77–92. 2086518

[B6] Casellini C. M. Parson H. K. Hodges K. Edwards J. F. Lieb D. C. Wohlgemuth S. D.. (2016). Bariatric surgery restores cardiac and sudomotor autonomic C-fiber dysfunction towards normal in obese subjects with type 2 diabetes. PLoS ONE 11:e0154211. 10.1371/journal.pone.0154211 27137224 PMC4854471

[B7] deJonge L. Moreira E. A. M. Martin C. K. Ravussin E. (2010). Impact of six-month caloric restriction on autonomic nervous system activity in healthy, overweight, individuals. Obesity 18, 414–416. 10.1038/oby.2009.408 19910943 PMC2882205

[B8] Eikelis N. Schlaich M. Aggarwal A. Kaye D. Esler M. (2003). Interactions between leptin and the human sympathetic nervous system. Hypertension 41, 1072–1079. 10.1161/01.HYP.0000066289.17754.49 12668587

[B9] Esler M. Jackman G. Bobik A. Kelleher D. Jennings G. Leonard P.. (1979). Determination of norepinephrine apparent release rate and clearance in humans. Life Sci. 25, 1461–1470. 10.1016/0024-3205(79)90371-0 513964

[B10] Flaa A. Aksnes T. A. Kjeldsen S. E. Eide I. Rostrup M. (2008). Increased sympathetic reactivity may predict insulin resistance: an 18-year follow-up study. Metabolism 57, 1422–1427. 10.1016/j.metabol.2008.05.012 18803948

[B11] Gerritsen J. Dekker J. M. TenVoorde B. J. Bertelsmann F. W. Kostense P. J. Stehouwer C. D. A.. (2000). Glucose tolerance and other determinants of cardiovascular autonomic function: the Hoorn Study. Diabetologia 43, 561–570. 10.1007/s001250051344 10855530

[B12] Grassi G. (2001). Renin-angiotensin-sympathetic crosstalks in hypertension: reappraising the relevance of peripheral interactions. J. Hypertens. 19, 1713–1716. 10.1097/00004872-200110000-00003 11593089

[B13] Grassi G. Dell'Oro R. Facchini A. Trevano F. Q. Bolla G. B. Mancia G. (2004). Effect of central and peripheral body fat distribution on sympathetic and baroreflex function in obese normotensives. J. Hypertens. 22, 2363–2369. 15614031 10.1097/00004872-200412000-00019

[B14] Grassi G. Dell'Oro R. Quarti-Trevano F. Scopelliti F. Servalle G. Paleari F.. (2005). Neuroadrenergic and reflex abnormalities in patients with metabolic syndrome. Diabetologia 48, 1359–1365. 10.1007/s00125-005-1798-z 15933859

[B15] Huggett R. J. Scott E. M. Gilbey S. G. Stoker J. B. Mackintosh A. F. Mary D. A. (2003). Impact of type 2 diabetes mellitus on sympathetic neural mechanisms in hypertension. Circulation 108, 3097–3101. 10.1161/01.CIR.0000103123.66264.FE 14676139

[B16] Huggett R. J. Hogarth A. J. Mackintosh A. F. Mary D. A. (2006). Sympathetic nerve hyperactivity in non-diabetic offspring of patients with type 2 diabetes. Diabetologia 49, 2741–2744. 10.1007/s00125-006-0399-9 16969648

[B17] Inzucchi S. E. Bergenstal R. M. Buse J. B. Diamant M. Ferrannini E. Nauck M.. (2012). Management of hyperglycaemia in type 2 diabetes: a patient-centred approach. Position statement of the American Diabetes Association (ADA) and the European Association for the Study of Diabetes (EASD). Diabetologia 55, 1577–1596. 10.1007/s00125-012-2534-0 22526604

[B18] Jennings G. Nelson L. Nestel P. Esler M. Korner P. Burton D.. (1986). The effects of changes in physical activity on major cardiovascular risk factors, hemodynamics, sympathetic function, and glucose utilization in man: a controlled study of four levels of exercise. Circulation 73, 30–40. 10.1161/01.CIR.73.1.30 3510088

[B19] Julius S. Valentini M. Palatini P. (2000). Overweight and hypertension: a 2-way street? Hypertension 35, 807–813. 10.1161/01.HYP.35.3.807 10720599

[B20] Kaiyala K. J. Prigeon R. L. Kahn S. E. Woods S. C. Schwartz M. W. (2000). Obesity induced by a high-fat diet is associated with reduced brain insulin transport in dogs. Diabetes 49, 1525–1533. 10.2337/diabetes.49.9.1525 10969837

[B21] Kim J. Montagnani M. Koh K. K. Quon M. J. (2006). Reciprocal relationship between insulin resistance and endothelial dysfunction. Molecular and pathophysiological mechanisms. Circulation 113, 1888–1904. 10.1161/CIRCULATIONAHA.105.563213 16618833

[B22] Lambert G. W. Straznicky N. E. Lambert E. A. Dixon J. B. Schlaich M. P. (2010). Sympathetic nervous activation in obesity and the metabolic syndrome-Causes, consequences and therapeutic implications. Pharmacol. Ther. 126, 159–172. 10.1016/j.pharmthera.2010.02.002 20171982

[B23] Lambert E. A. Straznicky N. Sari C. I. Eikelis N. Hering D. Head G.. (2013). Dyslipidemia is associated with sympathetic nervous activation and impaired endothelial function in young females. Am. J. Hypertens. 26, 250–256. 10.1093/ajh/hps016 23382410

[B24] Layman D. K. Boileua R. A. Erickson D. J. Painter J. E. Shiue H. Sather C.. (2003). A reduced ratio of dietary carbohydrate to protein improves body composition and blood lipid profiles during weight loss in adult women. J. Nutr. 133, 411–417. 12566476 10.1093/jn/133.2.411

[B25] Lee Z. S. Critchley J. A. Tomlinson B. Young R. P. Thomas G. N. Cockram C. S.. (2001). Urinary epinephrine and norepinephrine interrelations with obesity, insulin, and the metabolic syndrome in Hong Kong Chinese. Metabolism 50, 135–143. 10.1053/meta.2001.19502 11229419

[B26] Lien L. F. Brown A. J. Ard J. D. Loria C. Erlinger T. P. Feldstein A. C.. (2007). Effects of PREMIER lifestyle modifications on participants with and without the metabolic syndrome. Hypertension 50, 609–616. 10.1161/HYPERTENSIONAHA.107.089458 17698724

[B27] Machleidt F. Simon P. Krapalis A. F. Hallschmid M. Lehnert H. Sayk F. (2013). Experimental hyperleptinemia acutely increases vasoconstrictory sympathetic nerve activity in healthy humans. J. Clin. Endocrinol. Metab. 98, E491–E496. 10.1210/jc.2012-3009 23393176

[B28] Mackintosh R. M. Hirsch J. (2001). The effects of leptin administration in non-obese human subjects. Obes. Res. 9, 462–469. 10.1038/oby.2001.60 11500526

[B29] Mark A. L. (2013). Selective leptin resistance revisited. Am. J. Physiol. Regul. Integr. Comp. Physiol. 305, R566–R581. 10.1152/ajpregu.00180.2013 23883674 PMC3763044

[B30] Masuo K. Kawaguchi H. Mikami H. Ogihara T. Tuck M. L. (2003). Serum uric acid and plasma norepinephrine concentrations predict subsequent weight gain and blood pressure elevation. Hypertension 42, 474–480. 10.1161/01.HYP.0000091371.53502.D3 12953019

[B31] Matsuda M. DeFronzo R. A. (1999). Insulin sensitivity indices obtained from oral glucose tolerance testing. Diabetes Care 22, 1462–1470. 10.2337/diacare.22.9.1462 10480510

[B32] Matthews D. R. Hosker J. P. Rudenski A. S. Naylor B. A. Treacher D. F. Turner R. C. (1985). Homeostasis model assessment: insulin resistance and β-cell function from fasting plasma glucose and insulin concentrations in man. Diabetologia 28, 412–419. 10.1007/BF00280883 3899825

[B33] Ozata M. Ozdemir I. C. Licinio J. (1999). Human leptin deficiency caused by a missense mutation: multiple endocrine defects, decreased sympathetic tone, and immune system dysfunction indicate new targets for leptin action, greater central than peripheral resistance to the effects of leptin, and spontaneous correction of leptin-mediated defects. J. Clin. Endocrinol. Metab. 84, 3686–3695. 10.1210/jcem.84.10.5999 10523015

[B34] Parati G. Saul J. P. Castiglioni P. (2004). Assessing arterial baroreflex control of heart rate: new perspectives. J. Hypertens. 22, 1259–1263. 10.1097/01.hjh.0000125469.35523.32 15201539

[B35] Pricher M. P. Freeman K. L. Brooks V. L. (2008). Insulin in the brain increases gain of baroreflex control of heart rate and lumbar sympathetic nerve activity. Hypertension 51, 514–520. 10.1161/HYPERTENSIONAHA.107.102608 18158342

[B36] Rahmouni K. Haynes W. G. Morgan D. A. Mark A. L. (2003). Role of melanocortin-4 receptors in mediating renal sympathoactivation to leptin and insulin. J. Neurosci. 23, 5998–6004. 12853417 10.1523/JNEUROSCI.23-14-05998.2003 PMC6740337

[B37] Rahmouni K. Morgan D. A. Morgan G. M. Liu X. Sigmund C. D. Mark A. L.. (2004). Hypothalamic PI3K and MAPK differentially mediate regional sympathetic activation to insulin. J. Clin. Invest. 114, 652–658. 10.1172/JCI21737 15343383 PMC514588

[B38] Schlaich M. P. Grassi G. Lambert G. W. Straznicky N. Esler M. D. Dixon J.. (2009). European society of hypertension working group on obesity. Obesity-induced hypertension and target organ damage: current knowledge and future directions. J. Hypertens. 27, 207–211. 10.1097/HJH.0b013e32831dafaf 19155773

[B39] Sjostrom L. Peltonen M. Jacobson P. Sjöström C. D. Karason K. Wedel H.. (2012). Bariatric surgery and long-term cardiovascular events. JAMA 307, 56–65. 10.1001/jama.2011.1914 22215166

[B40] Snitker S. MacDonald I. Ravussin E. Astrup A. (2000). The sympathetic nervous system and obesity: role in aetiology and treatment. Obesity Rev. 1, 5–15. 10.1046/j.1467-789x.2000.00001.x 12119646

[B41] Solomon T. P. Haus J. M. Kelly K. R. Rocco M. Kashyap S. R. Kirwan J. P. (2010). Improved pancreatic β-cell function in type 2 diabetic patients after lifestyle-induced weight loss is related to glucose-dependent insulinotropic polypeptide. Diabetes Care 33, 1561–1566. 10.2337/dc09-2021 20200305 PMC2890359

[B42] Straznicky N. E. Lambert E. A. Lambert G. W. Masuo K. Esler D. M. Nestel P. J. (2005). Effects of dietary weight loss on sympathetic activity and cardiovascular risk factors associated with the metabolic syndrome. J. Clin. Endocrinol. Metab. 90, 5998–6005. 10.1210/jc.2005-0961 16091482

[B43] Straznicky N. E. Lambert E. A. Nestel P. J. McGrane M. Dawood T. Schlaich M.. (2010). Sympathetic neural adaptation to hypocaloric diet with or without exercise training in obese metabolic syndrome subjects. Diabetes 59, 71–79. 10.2337/db09-0934 19833893 PMC2797947

[B44] Straznicky N. E. Lambert E. A. Grima M. T. Eikelis N. Rischards K. Nestel P. J.. (2014). The effects of dietary weight loss on indices of norepinephrine turnover: modulatory influence of hyperinsulinemia. Obesity 22, 652–662. 10.1002/oby.20614 23997009

[B45] Tataranni P. A. Young J. B. Bogardus C. Ravussin E. (1997). A low sympathoadrenal activity is associated with body weight gain and development of central adiposity in Pima Indian men. Obes. Res. 5, 341–347. 10.1002/j.1550-8528.1997.tb00562.x 9285842

[B46] Vallbo A. B. Hagbarth K. E. Wallin B. G. (2004). Microneurography: how the technique developed and its role in the investigation of the sympathetic nervous system. J. App. Physiol. 96, 1262–1269. 10.1152/japplphysiol.00470.2003 15016790

[B47] Vaz M. Jennings G. Turner A. Cox H. Lambert G. Esler M. (1997). Regional sympathetic nervous activity and oxygen consumption in obese normotensive human subjects. Circulation 96, 3423–3429. 10.1161/01.CIR.96.10.3423 9396437

[B48] Vollenweider P. Randin D. Tappy L. Jéquier E. Nicod P. Scherrer U. (1994). Impaired insulin-induced sympathetic neural activation and vasodilation in skeletal muscle in obese humans. J. Clin. Invest. 93, 2365–2371. 10.1172/JCI117242 8200969 PMC294442

[B49] Watkins L. L. Surwit R. S. Grossman P. Sherwood A. (2000). Is there a glycemic threshold for impaired autonomic control? Diabetes Care 23, 826–830. 10.2337/diacare.23.6.826 10841004

[B50] Wu J. S. Lu F. H. Yang Y. C. Chang S. H. Huang Y. H. Chen J. J.. (2014). Impaired baroreflex sensitivity in subjects with impaired glucose tolerance, but not isolated impaired fasting glucose. Acta Diabetol. 51, 535–541. 10.1007/s00592-013-0548-9 24408773

[B51] Wulsin L. R. Horn P. S. Perry J. L. Massaro J. D'Agostino R. (2015). Autonomic imbalance as a predictor of metabolic risks, cardiovascular disease, diabetes, and mortality. J. Clin. Endocrinol. Metab. 100, 2443–2448. 10.1210/jc.2015-1748 26047073

[B52] Young C. N. Deo S. H. Chaudhary K. Thyfault J. P. Fadel P. J. (2010). Insulin enhances the gain of arterial baroreflex control of muscle sympathetic nerve activity in humans. J. Physiol. 588, 3593–3603. 10.1113/jphysiol.2010.191866 20643774 PMC2988520

[B53] World Health Organization (2006). Definition and Diagnosis of Diabetes Mellitus and Intermediate Hyperglycemia: Report of a WHO/IDF Consultation. Geneva.

[B54] Prospective Studies Collaboration (2009). Body-mass index and cause-specific mortality in 900,000 adults: collaborative analyses of 57 prospective studies. Lancet 373, 1083–1096. 10.1016/S0140-6736(09)60318-4 19299006 PMC2662372

[B55] Australian Bureau of Statistics (2015). National Health Survey: First Results Australia 2014-15. Canberra: ABS 2015 (ABS Catalogue No. 4364.0.55.001).

[B56] Yufu K. Okada N. Ebata Y. Murozono Y. Shinohara T. Nakagawa M.. (2014). Plasma norepinephrine is an independent predictor of adverse cerebral and cardiovascular events in type 2 diabetic patients without structural heart disease. J. Cardiol. 64, 225–230. 10.1016/j.jjcc.2013.12.009 24529506

